# Green synthesis of silver nanoparticles from *Manilkara zapota* leaf extract for the detection of aminoglycoside antibiotics and other applications†

**DOI:** 10.1039/d4ra01906g

**Published:** 2024-07-23

**Authors:** Khushboo Sahu, Ramsingh Kurrey, Ajai Kumar Pillai

**Affiliations:** a Govt. V. Y. T. Post Graduate Autonomous College Durg-491 001 Chhattisgarh India drajaipillai@gmail.com +917882 393644; b National Center for Natural Resources, Pt. Ravishankar Shukla University Raipur-492 010 Chhattisgarh India

## Abstract

Antibiotics of aminoglycoside (AMG) class, such as streptomycin (STR), have been widely used to treat infectious diseases caused by Gram-negative bacteria in livestock and humans. In this study, a selective and sensitive colorimetric probe for the determination of STR was proposed based on eco-friendly green synthesized AgNPs from the leaf extract of *Manilkara zapota*. The mechanism for the detection of STR is based on the electrostatic interaction of opposite charges between negatively charged silver nanoparticle-*Manilkara zapota* leaf (AgNP–MZL) and STR, causing an aggregation-induced characteristic shift of the SPR band (from 390 nm to 570 nm wavelength) of AgNP–MZL. The morphology, size distribution and optical properties of AgNP–MZL were characterized using UV/visible absorption spectroscopy, FTIR spectroscopy, XRD, DLS, zeta-potential measurements and TEM. The selective determination of STR was experimentally confirmed by performing controlled testing with other classes of antibiotics. To test the sensitivity level of this method, the ratio of these two *A*_390_/*A*_570_ absorbance wavelengths was selected to provide a linear concentration plot between 5 and 100 ng mL^−1^ STR. The LOD and LOQ were calculated to be 3.5 ng mL^−1^ and 26.8 ng mL^−1^, respectively. Good precision was evaluated with a standard deviation of 0.45 ng mL^−1^ and a relative standard deviation of 2.0% (intraday) and 2.42% (interday) at 10 ng mL^−1^ for 3 replicate measurements. Advantages of the green synthesis of AgNP–MZL include its eco-friendly nature and it is easy, efficient, cost effective and selective for the detection of the AMG class of antibiotics, *i.e.* STR, in agricultural and environmental samples.

## Abstract 2

Antibiotics of aminoglycoside (AMG) class, such as streptomycin (STR), have been widely used to treat infectious diseases caused by Gram-negative bacteria in livestock and humans.

## Graphical abstract

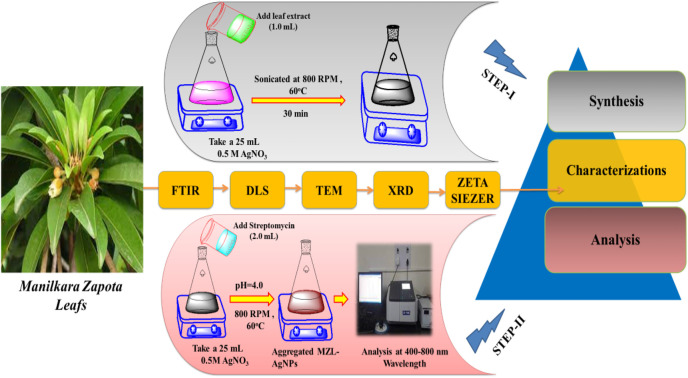

## 1 Introduction

**Scheme 1:**
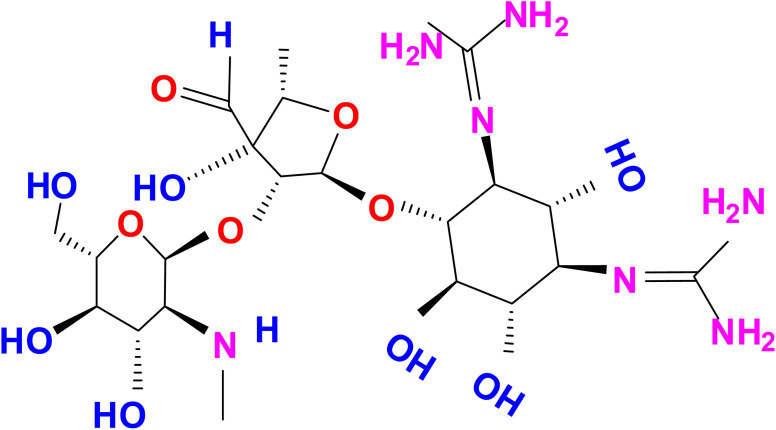
Chemical structure of aminoglycoside (AMG) class of antibiotic (STR).

Streptomycin (STR) is a water-soluble aminoglycoside antibiotic (AMG) with the chemical name d-streptamine, *O*-2-deoxy-2-(methylamino)-α-l-glucopyranosyl-(1→2)-*O*-5-deoxy-3-*C*-formyl-α-l-lyxofuranosyl-(1→4)-*N*,*N*1-bis(aminoiminomethyl)-sulfate (2 : 3 salt) (molecular formula: (C_21_H_39_N_7_O_12_)_2_·3H_2_SO_4_) and used to treat Gram-negative bacteria infections (Scheme 1).^1^ The antibiotic STR discovered in the 1950s was first used to control bacterial pathogens of human diseases and then used to control bacterial pathogens of plants, including those that cause rice diseases. STR was discovered soon after penicillin was introduced into medicine, making it the second most therapeutically useful antibiotic in the field of medicine. It provided the first effective cure for tuberculosis, tuberculosis meningitis and a range of other infections caused by pathogenic Gram-negative bacteria.^2^ STR is a broad-spectrum aminoglycoside antimicrobial agent obtained from *Streptomyces griseus* for Gram-negative bacterial infection treatment and is used not only in human health care but also in agriculture and veterinary medicine. Currently, the AMG class of antibiotics is frequently used in animal husbandry, food and agriculture. The excess of STR could result in the presence of STR residues in animal-derived food products, causing serious side effects on human health, such as allergic reactions, loss of hearing and toxicity to the kidneys.^3^ Moreover, because of its high water solubility, STR residues in the aquatic environment are difficult to remove completely. Thus, an efficient and accurate detection of STR in water and vegetable samples is essential for environmental monitoring and to protect human health.^4^ To ensure food safety and quality control, different regulations have been established for the AMG class of antibiotics in aquatic environments in the world, such as EC-2011, CDC-2015, ECDC-2015a, EFSA-2015, EMA-2015a and EC-2015b,c.^5–7^ Under these regulations, specific rules for the organized control of environmental products intended for human consumption are established, and a new procedure for the determination of AMG class of antibiotics such as STR residue in different sources, *e.g.* vegetable, food and water, are recommended. The detected concentrations generally range from ng L^−1^ to μg L^−1^ according to the aqueous environment matrices.^8^ Considering the hazard of STR excess, most countries have set a standard for STR residues in animal products.^9^ Although the use of STR has been banned in many countries, applications on a small scale still continue. For example, in 1998, apple trees in Germany were treated for reblight with 72 kg of STR applied in one growth season.^10^ Therefore, the development and application of nanoprobes for the sensitive and selective detection of STR is in significant demand to ensure human health as well as food quality and safety.

The available methods for veterinary drug detection involve priority approaches such as liquid and gas chromatography, microbiological assays, microplate enzyme-linked immunosorbent assay (ELISA) and immunochemical assays.^8–12^ The residue levels of STR are predominantly detected by high-performance liquid chromatography, liquid chromatography-mass spectrometry, and gas chromatography-mass spectrometry. Enzyme-linked immunosorbent assay, fluorescence immunoassay, and radioimmunoassay have also been used to detect STR residues; however, cross-reactions can prevent efficient determination of the target analyte.^8–12^ The latter has attracted special interest due to its high specificity and sensitivity combined with the fact that it does not require expensive complicated equipment. UV-Vis spectrophotometry dominates over other techniques in the actual practice of STR analysis. UV-Vis is a simple and rapid technique for the determination of STR although the selectivity of the method is poor due to the use of chromophoric reagents. Therefore, an alternative method is required that should be simple, selective, label-free and low cost for the determination of STR using green nanotechnology from different types of vegetable, water and soil samples.

Recently, green nanotechnology has emerged as a rapidly growing field with numerous applications in science and technology for eco-friendly green synthesis of new materials.^13^ Green nanotechnology has the potential to change science, the economy, and daily life in the twenty-first century.^14,15^ Potential uses of green synthesized nanomaterials include *in vivo* and *in vitro* biomedical research and applications. Among the various plant organs, *Eucalyptus globulus* Labill and *Eucalyptus robusta* Sm are used for the synthesis of metal nanoparticles (MNPs), such as iron, silver, gold and titanium.^16^ A plant organ has shown pharmacological activity, especially in cancer and inflammation as well as antioxidant, antimicrobial and biodegradability.^17^ AgNPs are usually used for a wide range of applications, such as the removal of heavy metals, dyes and antibiotics from water sources. Particles up to 100 nm in size are commonly referred to as nanoparticles.^18^ Nanoparticles can be synthesized by applying physical, chemical and biological methods. Extreme circumstances, including high pressure, energy, temperature, and hazardous substances, are occasionally necessary for physical and chemical techniques, whereas biological techniques are economical, environmentally benign and depend on the utilisation of microbes, enzymes, and plant extracts.^19,20^ Green synthesized silver nanoparticles (AgNPs) have proved to be most effective because they have good antimicrobial efficacy against most pathogenic bacteria. It has been reported that AgNPs are nontoxic to humans and most effective against bacteria, viruses and other eukaryotic microorganisms at low concentrations, without any side effects.^21^ Compounds such as terpenoids, flavonoids, various heterocyclic, polyphenols, reducing sugars and ascorbate are directly involved during the green synthesis process, which is one of the key advantages of this method. Other than this, all compounds are also responsible for the reduction and formation of stabilized AgNPs. Thus, green-synthesized AgNPs play a major role in the fields of nanotechnology and nanomedicine.^22–24^ In the last few years, several studies have been carried out for the green synthesis of nanoparticles from various plant parts, such as fruit extract of *Cynometra ramiflora*, rind of *Persea americana*, seeds extract of *Punica granatum*, and flower extract of *Avicennia marina*, for degradation and biological applications.^25^ To the best of our knowledge, there are very few methods that have been used to detect antibiotics using green synthesis of AgNPs from plant extract. In these ways, we developed and designed a new method for the green synthesis of AgNPs from the leaf extract of the *Manilkara zapota* plant.

In the present work, the leaf extract of the *Manilkara zapota* (MZ) plant was used for the green synthesis of AgNPs under optimized conditions, such as the volume of *Manilkara zapota* leaf (MZL) extract, the concentration of silver salt and temperatures. The novel nanosensor, *i.e.* AgNP–MZL, acted as a sensing probe for the selective and sensitive detection of the AMG class of antibiotics, such as STR using UV-Vis in different types of agricultural and environmental water and soil samples. Antioxidant and antibacterial activities were also studied to ensure the good efficiency of green nanomaterials. The LOD values obtained were far below those of the MRL levels of STR in the water and vegetable samples. The results showed that the AgNP–MZL probe is rapid, selective, and sensitive towards STR and thus could serve as the basis for novel assessments to ensure food safety and human health.

## 2 Experimental design

### 2.1 Apparatus

The UV-Vis absorbance spectroscopy was carried out using a double beam spectrophotometer (type Cary-60, Agilent Technologies, USA) with 1 cm of cell made up of quartz. The infrared (IR) spectra of the biosynthesized AgNP–MZL were obtained using an attenuated total reflectance-Fourier transform infrared spectrometer (ATR-FTIR) (type-Nicolet-iS10 Thermo Scientific, USA). Alternatively, the size and shape of AgNP–MZL with and without the addition of an AMG class of antibiotics, such as STR, were determined using a transmission electron microscope (TEM) (Jeol, IET-2200FS) with a 100 kV accelerating voltage in the presence and absence of material components. X-ray diffraction (XRD) analysis was recorded in the range of 20°–60° using a diffractometer (Bruker, Germany) with Cu K, radiation along with an accelerating voltage of 40 kV at a scanning rate of 1° per minute.

### 2.2 Chemicals, reagents and standard solutions

All chemicals used were of analytical grade, so no further purification by applying the chemical method was needed. Silver nitrate (AgNO_3_) was used as a precursor, and sodium borohydride was used as a reducing agent. Sodium hydroxide (NaOH) and hydrochloric acid (HCl) were purchased from ACS Reagent, 97%, Sigma-Aldrich for the preparation of AgNP–MZL. Different species, such as dicholorovos, thiochloprid, monocrotophos, cypermethrin, acetamethrin, bifenthrin and streptomycin, were purchased from Hi-Media Laboratories Pvt. Ltd., Mumbai, India. A stock standard solution (1000 ng mL^−1^) of all AMG classes of antibiotics was prepared by dissolving an appropriate amount of the substance in ultrapure water. Further serial dilution in the range of 5–100 ng mL^−1^ was prepared from 1000 ng mL^−1^ of stock standard solution. By dissolving 0.017 g of AgNO_3_ in 100 mL of Milli-Q water, a 1.0 mmol L^−1^ aqueous solution of AgNO_3_ was prepared and used for the green synthesis of silver nanoparticles (AgNPs).

### 2.3 Collection and preparation of plant materials

MZL was collected from the Raipur District of Chhattisgarh using clean polyethylene bags and washed several times with distilled water. The samples were dried, crushed, or ground in a mill or with a pestle and mortar, after which the sample was homogenized. After this, a 5.0 g powder sample was added into 50 mL Milli-Q water (H_2_O) as an extracting medium in a round bottom flask and heated at 80 °C with continuous stirring at 400 rpm for 1 hour in Digital Spinot. Subsequently, the sample solution was cooled at room temperature and then filtered using 0.45 μm Whatman filter paper. The samples were stored at 4 °C overnight and cooled, and a resulting brown colour leaf extract was obtained. Finally, the prepared leaf extract was used for the synthesis of AgNP–MZL.

### 2.4 Green synthesis of AgNP–MZL as a carrier material

AgNP–MZL was prepared through nanoprecipitation according to a previously reported methodology with a few modifications.^26^ The formation of AgNP–MZL was carried out using different volumes of MZL extract (1.0 to 2.0 mL) and silver salt (AgNO_3_) concentrations of 0.5 M and 5.0 M, respectively. The silver precursor solution was added to the plant extract of MZL in a ratio of 4 : 1; the resulting mixture was sonicated at 800 rpm and 60 °C for 30 min in Tarson's Digital Spinot. After some time, the colour of the solution mixture changed to yellowish brown. Note the reading at which the solution completely turns dark blackish brown in colour. The dark blackish brown indicates the formation of AgNP–MZL, which is confirmed by the spectroscopic studies. Finally, AgNP–MZL was stored under refrigeration until their characterization, detection of the AMG class of antibiotic (STR) and evaluation of antimicrobial and antibacterial activity were performed. Effective AgNP–MZL synthesis was achieved by optimizing the following parameters:

(a) Volume of MZL ranging from 1.0 to 2.0 mL.

(b) Concentration of silver metal ions ranging from 0.5 to 5.0 M.

(c) Concentration of AgNP–MZL ranging from 0.05 to 2.0 mM.

(d) Temperature ranging from 25 to 80 °C.

(e) Time of incubation ranging from 0 to 25 min. The reaction mixtures were periodically monitored in the range of 200–800 nm using a UV-Vis spectrophotometer to detect the formation of AgNP–MZL.

### 2.5 Sample collection and preparation for analytical applications

Different environmental water samples were collected in cleaned polyethylene bottles from rural areas of the state of Chhattisgarh, India. The collected samples showed a clear appearance and no suspended particles. The bottles were washed with a solution of 1.0% v/v alkaline detergent under an ultrasonic bath for 30 minutes, rinsed several times with ultrapure water and filled with the same water sample to eliminate contamination of the container.^27^ Water samples collected with these bottles were carefully filled to the brim to avoid trapping air. After filling the bottles, they were sealed with Teflon-lined screw caps, kept on ice, and transported to the laboratory before 24 h; they were stored at 3 °C until the spectrophotometric analysis.

**Fig. 1:**
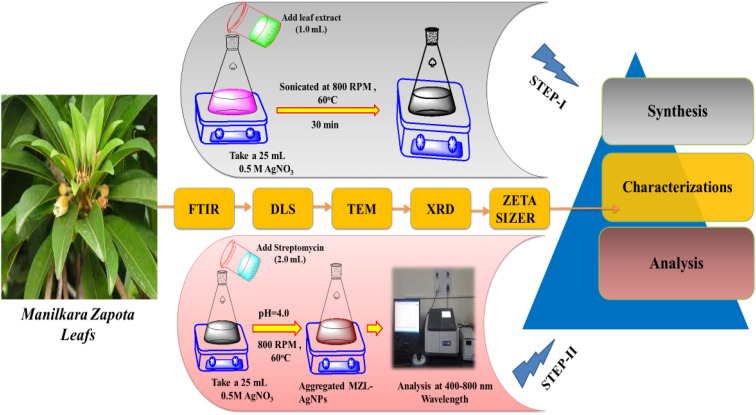
Schematic diagram for the determination of the AMG class of antibiotic (STR) using the green synthesis of AgNPs by MZL extract (STEP-I and STEP-II).

Different agricultural (potato, tomato and green beans) and environmental soils were collected using clean polyethylene bags and washed several times with distilled water. The samples were crushed, or ground in a mill or with a pestle and mortar, after which the sample was homogenized. Extracting the STR from agricultural samples, such as potatoes, tomatoes and green beans, can be performed using Soxhlet extraction (SOX-606 Automatic Soxhlet Extractor). The next day, a 1.0 g powder sample was refluxed by adding 10 mL of ethanol : water (4 : 1 ratio) for 4 hours using an automatic Soxhlet apparatus.^28^ The sample extract was centrifuged at 1613 rcf with a 10 mm radius of rotor for 15 min to remove any debris present in the sample before filtration using 0.45 μm pore size of Whatman filter paper. Finally, the pre-concentrated agricultural samples were used for the quantitative analysis of STR using AgNP–MZL by UV-visible spectrophotometry. Fig. 1 displays a schematic diagram for the determination of STR using green synthesis of AgNPs by MZL extract (STEP-I).

### 2.6 Colorimetric method for the detection of the AMG class of antibiotics

In the present work, the quality control and quality assurance methods were carried out according to EURACHEM guidelines.^28^ The sensing ability of AgNP–MZL against STR was examined. For this, a 20 μL AgNP–MZL probe solution was introduced by increasing the concentrations of STR from 5 to 100 ng mL^−1^. The pH of the sample solution was adjusted to 4.0 using 1 N HCl and 1% NaOH solutions. The volume up to the mark with water was made up, and the absorbance at 390 nm was analyzed using the blank solution as a reference. The color of the sample solution was changed from dark blackish brown to dark brownish color, indicating the aggregation of AgNP–MZL with the AMG class of antibiotics, such as STR. Finally, STR-treated AgNP probe solutions were used to record UV-Vis absorption spectra in the range of 400–800 nm wavelengths under the optimum conditions (Fig. 1, STEP-II).

Based on the above procedure, the calibration curve was prepared by utilizing the respective localized surface resonance band ratio (LSR) at *A*_390_/*A*_570_ nm obtained for the minimum and maximum concentration ranges of STR (5, 10, 15, 20, 40, 50, 80, and 100 ng mL^−1^). The calibration curve is described by the equation *Y* = *MX* + *C*, where *X* represents the concentration of STR and *Y* represents the absorbance value. The linear least square equation obtained from this curve was used for the quantitative determination of the AMG class of antibiotic (STR) from agricultural samples, such as fruit and vegetable samples. Intra-day and inter-day repeatability were calculated with three replicates. Recovery was evaluated using blank sample water spiked with 10 and 50 ng mL^−1^. The limits of detection (LOD) and limits of quantification (LOQ) were calculated from the mean and standard deviation (SD) of eight blank measurements with a 95% confidence limit.

### 2.7 *In vitro* antioxidant and antimicrobial activities

The *in vitro* antioxidant activity of the seed extract of *Manilkara zapota* was determined by the entrapment test of the 1,1-diphenyl-2-picrylhydrazyl radical (DPPH) assay based on the method described in the reported articles with a few modifications.^26^ For this analysis, 50 mL of leaf extract of *Manilkara zapota* was taken, placed into a 100 mL volumetric flask and then mixed with 50 mL of ethanol and 25 mL of DPPH solution. After that, the extract was incubated in the dark at room temperature for 1 h. The content of biochemical (quercetin) in the sample was the same as that of the control according to the load rate of the target compound. The absorbance of the mixture was recorded at 570 nm. The absorption band of the resulting solutions against the corresponding blank sample was measured using the UV-Vis spectrophotometric method.

i (%) scavenging = (*A*_b_) − (*A*_s_)/(*A*_b_) × 100,

where (*A*_b_) is the absorbance of the blank sample and (*A*_s_) is the absorbance of the standard.

Next, the antibacterial activity was carried out against *Escherichia coli*, *S. aureus*, and *Enterococcus faecalis* using the disc diffusion method.^29^ For this study, a set of sterile discs was impregnated with three concentrations of AgNP–MZL, *i.e.*, 5 μg per disc, 10 μg per disc and 20 μg per disc in μL of volumes. After that, the discs were smoothly incubated in a reversed position for 1 day at 37 °C after the preparation of the cultural plates. After the incubation period, the susceptibility of the test microorganisms was determined by the diameter of the zone inhibition for statistical evaluation.

## 3 Results and discussion

### 3.1 Characterization of AgNP–MZL

**Fig. 2:**
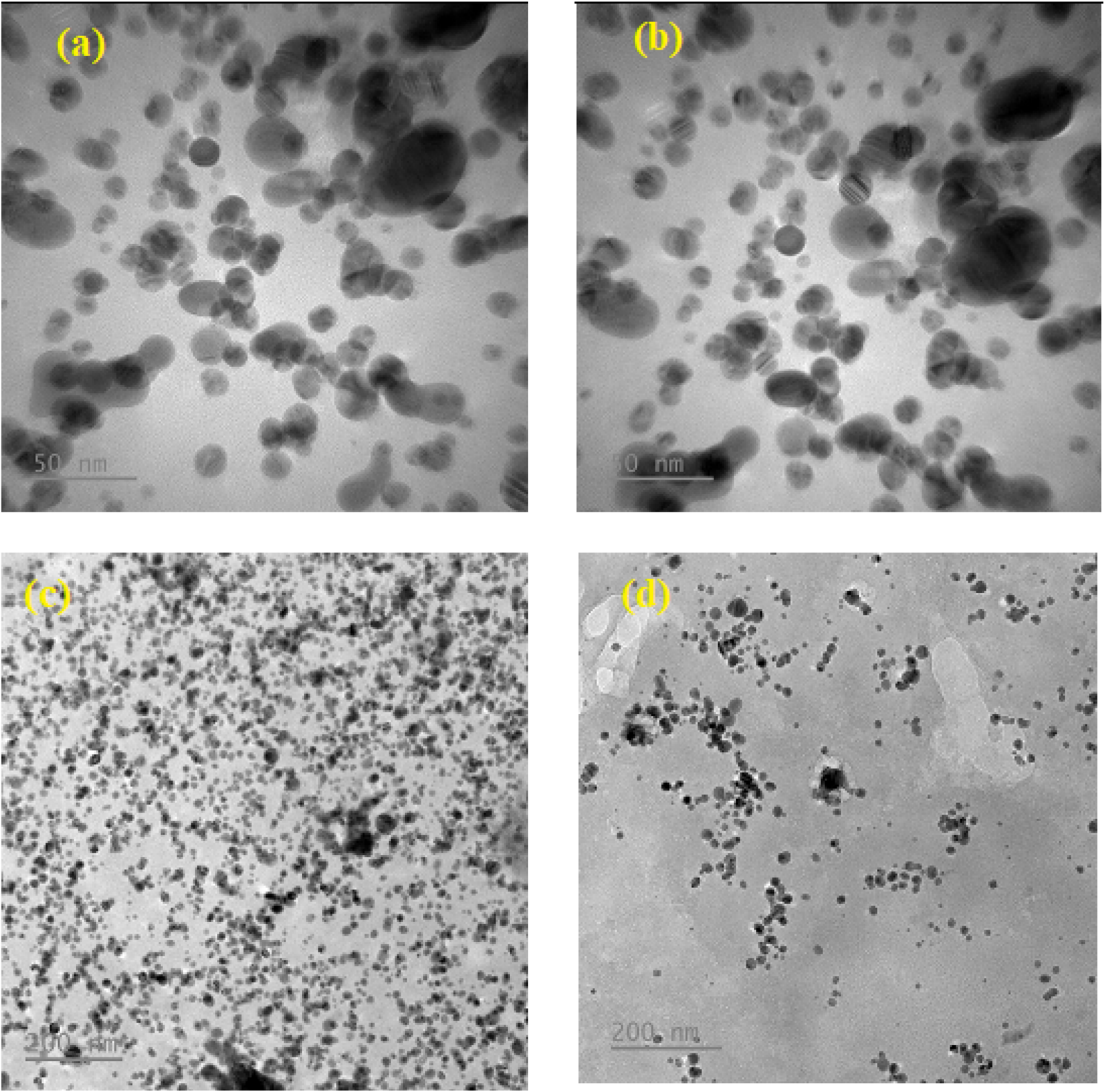
Two magnifications of ×50 and ×200 nm of the TEM image of (a and b) green synthesized AgNP–MZL and (c and d) green synthesized AgNP–MZL binding with STR antibiotic, respectively.

**Fig. 3:**
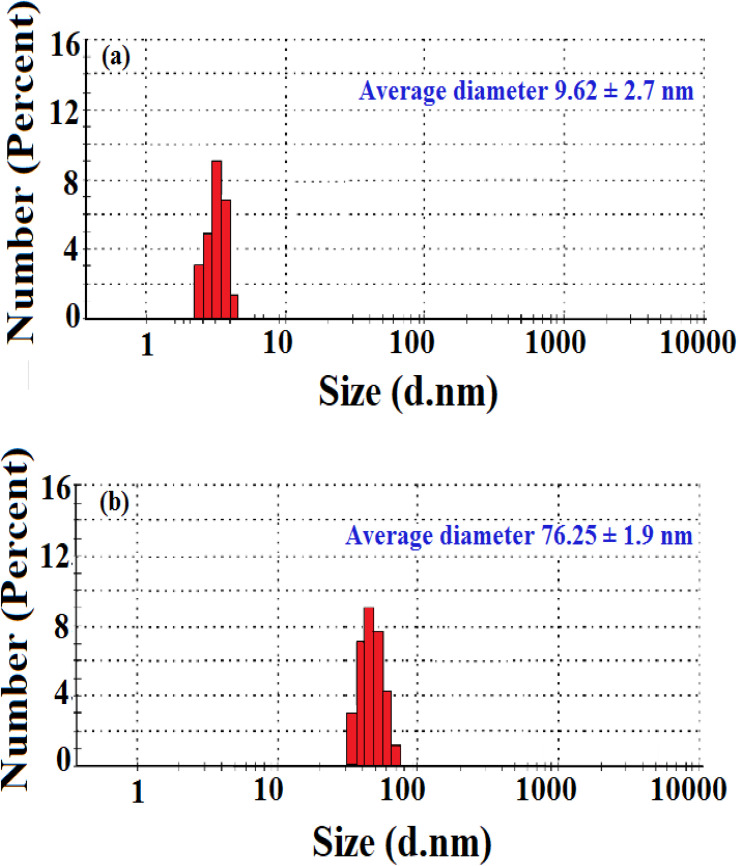
Size distribution of green synthesized AgNP–MZL (a) and green synthesized AgNP–MZL binding with the AMG class of antibiotic (b).

The shape and morphology of the synthesized nanoparticles were identified through scanning electron microscope analysis. The nanoparticles were examined under various magnifications of ×50, and ×200 TEM images of the green synthesized AgNP–MZL, as shown in Fig. 2. The results demonstrated a relatively clustered shape. Accumulation of two or more reducing moieties bound on the surface of the preformed nuclei of particles may have contributed to the formation of elongated large nanoparticles. The TEM images clearly show that the freshly prepared green synthesized AgNP–MZL were spherically shaped, monodispersed in nature, and well dispersed in aqueous solution with an average particle size of 10.41 nm, as shown in Fig. 2(a) and (b), in the absence of AMG antibiotic, *i.e.*, STR. However, as illustrated in Fig. 2(c) and (d), the dynamic size of AgNP–MZL dramatically increased in the presence of AMG antibiotic (STR), with a diameter of about 60.53 nm. In this investigation, the DLS curves indicated the particle size distribution obtained from green synthesized AgNP–MZL in the presence and absence of STR. It is always the case that the size distribution evaluated through DLS is better than that obtained from UV-Vis spectroscopy. The DLS analysis of dispersed AgNP–MZL and aggregated AgNP–MZL with STR showed particle sizes of 9.62 ± 2.7 nm and 76.25 ± 1.9, respectively (Fig. 3(a) and (b)). Nanoparticles smaller than TEM may be the reason for particle agglomeration.

**Fig. 4:**
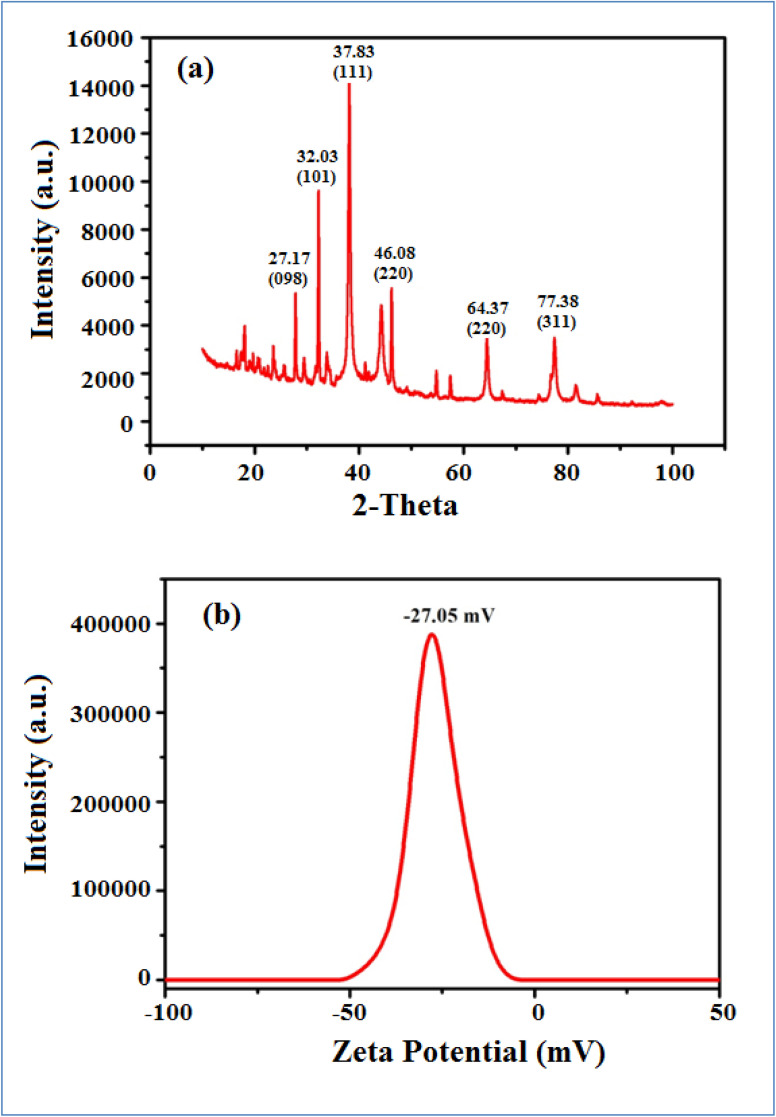
XRD pattern (a) and zeta potential (b) of green synthesized AgNPs by MZL extract.

Furthermore, the size and size distribution of the green synthesized AgNP–MZL were obtained using a Zetasizer after dispersion in water at a temperature of 25 °C. The measurement of zeta potential depends on the movement of nanoparticles under the influence of an applied electric field. This movement depends on the surface charge and the local environment of the particle.^30^ The stability of the green-synthesized AgNP–MZL was at −27.05 mV, which indicates excellent stability, good dispersion and the presence of various plant species, such as secondary metabolites and phytochemicals, as capping or reducing agents.^30^ The results are shown in Fig. 4(b). In addition, the X-ray diffraction (XRD) pattern clearly revealed that the AgNP–MZL formed was crystalline, displaying structural information. The XRD spectrum analysis indicated two diffraction peaks at 27.17°, 32.03°, 37.83°.46.08°, 64.37° and 77.38°. These diffraction lines are obtained at 2*θ* angles, which can be indexed as (98), (101), (111), (200), (220) and (311) to form the face center cubic (FCC) structure of silver (Fig. 4(a)). The (111) plane was chosen as the average crystalline size of AgNPs because it showed an intense peak from the XRD analysis. These findings have been verified by the literature report on measuring the shape and size of green synthesized AgNP–MZL, as well as the size distribution of nanoparticles.^30^

**Fig. 5:**
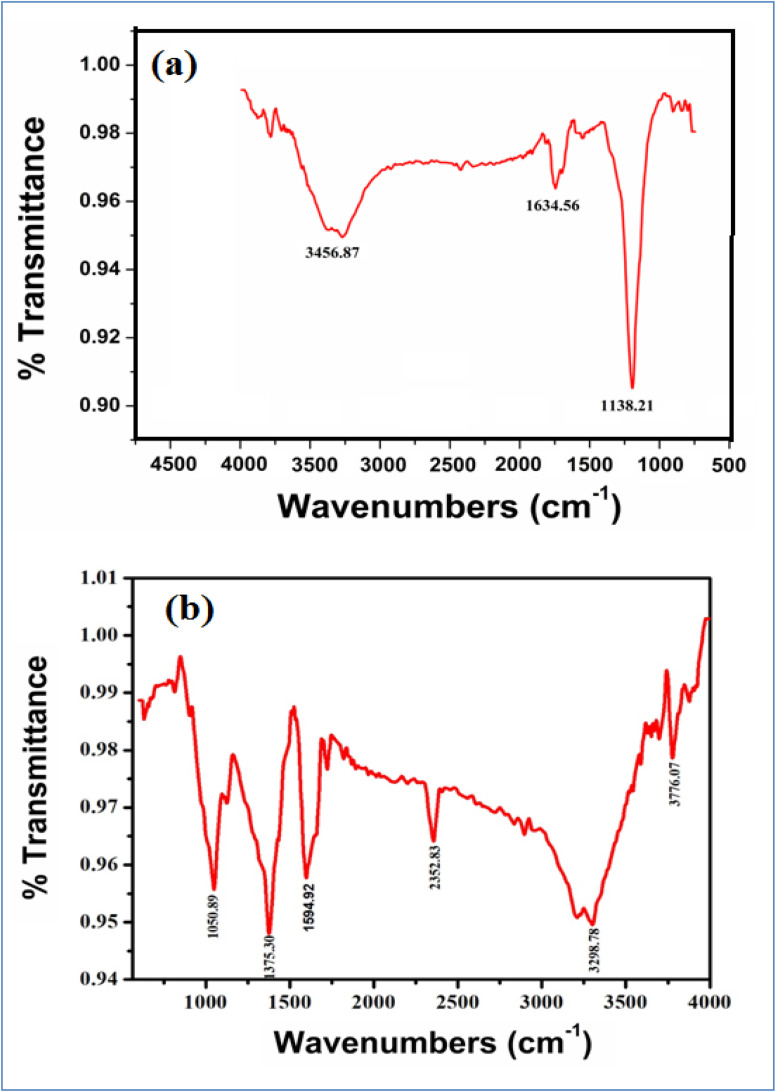
FTIR spectra of green synthesized dispersed AgNPs from MZL extract (a) and aggregated AgNP–MZL with AMG antibiotic (STR) (b).

Fig. 5(a) and (b) and Table 1 show the FTIR spectra of green synthesized AgNPs from MZL extract, and green synthesized AgNP–MZL aggregate with AMG antibiotic, *i.e.*, STR. FTIR absorption spectra were recorded for green synthesized AgNPs by *Manilkara zapota* leaf extract. The purpose was to detect the major phytochemicals responsible for the reduction of the metal and stabilization during the synthesis of AgNPs. The FTIR spectrum in the range of 3700–3200 cm^−1^ represents O–H stretching and H-bonded alcohols and phenol. The peak found around 2260–2100 cm^−1^ shows a stretch for (–C=C–) bond, which corresponds to alkenes, while the peak found around 1650–1580 cm^−1^ shows the bond for (N–H) bending, which corresponds to the primary amine, and the peaks in the range of 1250–500 cm^−1^ are due to C–H bending in the aromatic ring, =C–H and =CH_2_ out-of-plane bending, –OH bending, C–H deformation *etc.* The position of the peak above shifted to a slightly higher wavenumber after the reduction of Ag^+^ with phytochemicals species, which are in the presence of the MZL extract. The strong and intense peak at 1138.21 cm^−1^ owing to the C–O stretching vibrations disappeared after the interaction of phytochemical species with Ag^+^. In addition, vibrational peaks from the fingerprint region appeared at 1634.56 cm^−1^ for the (N–H) bending of the primary amine, as shown in Fig. 4(a), owing to the interaction of aromatic rings of plant species with Ag^+^. The observed strong and sharp infrared peaks were compared with standard values to identify the functional groups. The FTIR spectra of the AMG class of antibiotics, such as STR, changed greatly upon combination with AgNP–MZL, as displayed in Fig. 4(b). In the case of STR, the band at 1138.21 cm^−1^ was shifted to a higher wavelength at 1373.30, which suggests that the antibiotic interacts with the AgNP–MZL through its carbonyl group (C=O). Furthermore, the peak of the primary amine at 3456.87 cm^−1^ shifted to 3298.72 cm^−1^ after the combination with nanoparticles, indicating that the amine functional group was involved in the interaction with the surface of the phytochemical containing AgNP–MZL (Fig. 5(b)). The results of the FTIR suggest that the functional groups of the AMG class of antibiotics (STR) could be involved in the interaction by hydrogen bonds with phytochemical species.^30,31^

**Table 1:** Functional groups with their wavenumber (cm^−1^) values of MZL extract, AgNP–MZL, pure STR and AgNP–MZL bind with antibiotics

S. no.	Name	Functional group	Peak assignment	Present method	Reported spectral data of pure compound
				Peak (cm^−1^)	Peak (cm^−1^)
1	MZL extract	Intermolecular H-bonded of phenolic compounds	O–H stretching vibration	3315.71	3303.08
		Alkanes	C–H stretching vibration	2928.12	2925.44
		Carboxalate	RCOO– stretching vibration	1456.33	1406.20
		Amino acid	NH bending vibration	1638.76	1613.75
		Phenols/alcohols	C–O stretching vibration	1101.31	1047.37
2	AgNP–MZL	Interaction of the MZL extract species and silver metal	All species and Ag^+^	3456.56	3301.39
		Alkanes	C–H stretching vibration	2950.11	2927.56
		Carboxalate	RCOO– stretching vibration	1355.39	1381.77
		Amino acid	NH bending vibration	1634.56	1619.01
		Phenols/alcohols	C–O stretching vibration	1014.22	1056.83
3	AMG antibiotic (STR)	Alcohol	O–H stretching and H-bonded	3315.81	3563.98
		Alkanes	–C=C–	1556.43	1406.29
		Amine	N–H	3456.87	3448.72
		Alkene	C–H	2951.19	2948.51
		Ketone	C–O stretching vibrations	1125.23	1056.83
4	AgNP–MZL bind with STR	Alcohol	O–H stretching and H-bonded	3298.78	3498.77
		Alkanes	–C=C–	2352.83	2455.81
		Carbonyl		1598.82	1698.88
		Amine	N–H	3298.78	3256.85
		Ketone	C–O stretching vibrations	1050.89	1250.50

### 3.2 Screening of the green synthesized AgNP–MZL for detection of the AMG class of antibiotic

**Fig. 6:**
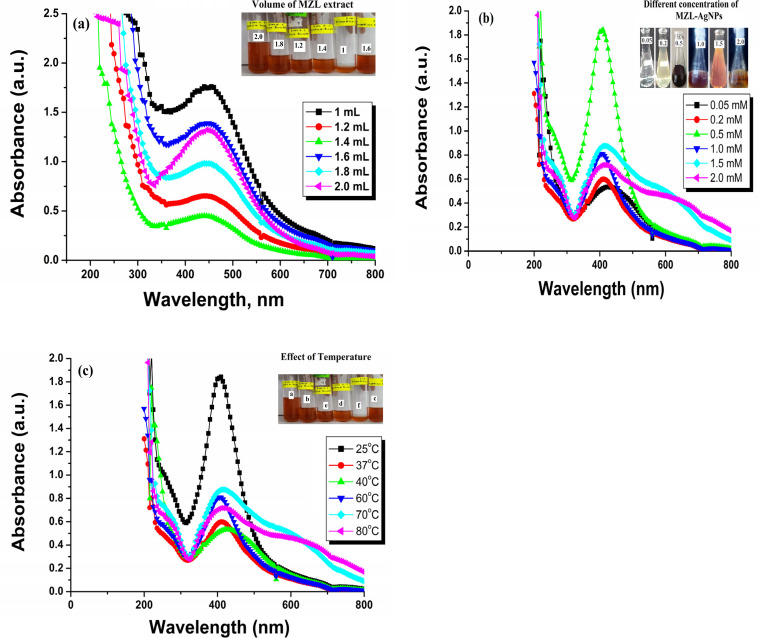
Optimization of the green synthesized AgNP–MZL: effect of volume of MZL extract (a), effect of concentration of the green synthesized AgNP–MZL (b) and effect of temperature (c).

**Fig. 7:**
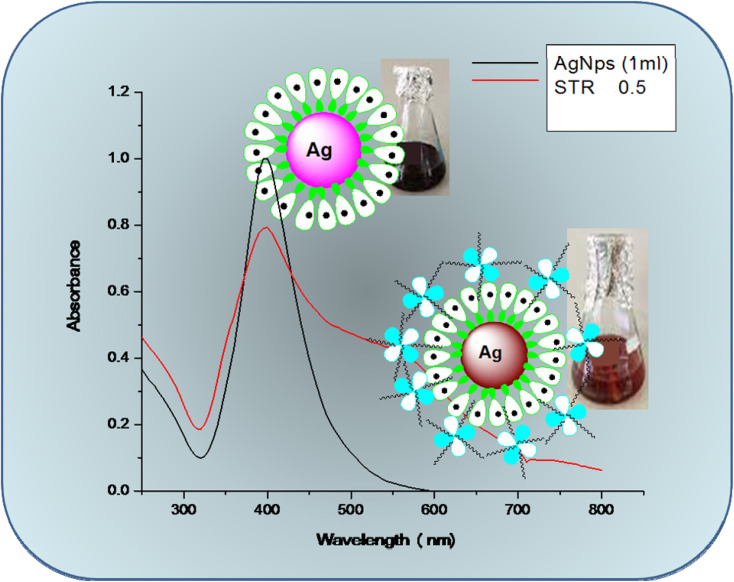
Glass-vial containing UV-visible spectra of green synthesized AgNPs from MZL extract (black colour) and AgNP–MZL bind with AMG class of antibiotic (red colour).

UV-Vis spectroscopy is an easily available and most effective approach for the identification of NP formation.^32^ UV-visible spectroscopy was used to examine the bio-reduction of metal ions (M^+^) in the solution. The mixture of metal (silver) salt with different volumes of plant extract was analyzed for its spectra and *λ*-max. A small amount of material was placed in a quartz cuvette with distilled water as a reference, and the wavelength was measured using a spectrophotometer in the range of 200–800 nm. In the present investigation, an effort was made to evaluate the potential of the MZL extract to reduce silver salts, resulting in the formation of AgNP–MZL. AgNPs were formed at room temperature when various amounts of MZL extract, such as 1.0, 1.2, 1.4, 1.6, 1.8, and 2.0 mL, were added dropwise in solutions of AgNO_3_ that were made (0.5 mM). For silver, a progressive colour change from pale green to dark blackish-brown was observed, signifying the formation of NPs. A slight colour change was observed when different MZL extract quantities were used to react with a constant volume (2.0 mL) of 0.5 mM silver salt solution. When combined with 2.0 mL of MZL extract, a 0.5 mM salt solution showed a more noticeable colour change than the other quantities. The results are shown in Fig. 6(a)–(c). The colour change is due to the collective vibrations of the charged particles present on the surface of the nanoparticles and the resonance. NPs need reducing agents and stabilizers for their synthesis and to prevent their aggregate formation after synthesis.^33^ The phytochemicals present in plant extracts have a reducing potential because they are responsible for NP formation. Plants such as *Manilkara zapota* have several phenolic compounds with evident antioxidant activity due to the availability of H^+^ ions. These ions are also responsible for the reduction of metal ions into nanoparticles.^34^ Green synthesis of AgNPs is also well thought out as less toxic to the environment in comparison to chemical synthesis. Amutha and Sridhar also confirmed the green synthesis of NPs using plant extract and salt solution.^35^ A typical plasmon resonance band at 390 nm is observed in the UV-Vis absorption spectra of the AgNPs synthesized using MZL extract, indicating the formation of AgNPs. In addition, the UV-Vis spectrum of the leaf extract was analyzed, and it did not show any absorption band in the range of 200–800 nm (Fig. 7).

Green synthesized AgNP–MZL was chosen for the selective detection of the AMG classes of antibiotics, such as STR, owing to its bio-chemical stability, antioxidant, and antiangiogenic activity; antibacterial activity; and anticancer and catalytic properties. In addition, the high surface area-to-volume ratio of AgNP–MZL is found to be good for surface interactions with STR, causing the shift of the LSPR absorption band in colorimetric analysis. The LSPR absorption band of the NPs was shifted to a higher wavelength from 390 nm to 570 nm when STR was added into the NPs due to the aggregation of particles (Fig. 7). In addition, the dark blackish-brown coloured monodispersed AgNP–MZL in aqueous medium turned dark brownish color after the addition of STR only and not with the addition of other tested antibiotics. Therefore, the AgNP–MZL was used for the selective detection of the AMG class of antibiotics in environmental, agricultural and soil samples using the UV-Vis spectrophotometric method.

In addition, green synthesized AgNPs were developed using MZL extract, which acted as stabilizing agents for the respective nanoparticle solutions. The reducing agent acts as a stabilizing agent without any additional chemicals. Because the agent is biological in origin, the synthetic process is green and eco-friendly. Bioactive compounds were detected in the leaf extracts of MZ. Their presence in this extract could explain the antibacterial activities observed.^34^ The significant reduction in the Gram-positive *S. aureus* viability at constant metal content confirms that the surface functionalization of Ag nanoparticles is an important approach to counter them. Furthermore, the antibacterial impact was more significant towards *E. coli* than *S. aureus* and *E. faecalis*. This differential impact is possibly due to the differences in the cell walls of both bacterial strains, which needs to be further confirmed. Very clear zones were observed around the wells loaded with NPs and plant extract, which proved the effectiveness of the NPs. Tamokou *et al.* proposed that the leaf extracts of MZ presented more significant activity than other parts of this plant.^36^ It displayed significant activity against 10 ± 3.4 and 6 ± 1.9 of the Gram-negative bacteria tested for *E. coli* and *S. aureus*, respectively. Similarly, the green synthesized AgNP–MZL was evaluated for *in vitro* antioxidants using DPPH as a substrate. The DPPH scavenging activity of green synthesized AgNP–MZL was 48.65% at a concentration of 50 ng mL^−1^. The characteristic property of DPPH is that it is a stable free radical and accepts electrons or hydrogen from green synthesized AgNP–MZL during the activity. Therefore, these results indicate that the green synthesized AgNP–MZL has excellent physical and chemical properties for the selective detection of STR in real environmental, agricultural and soil samples.

### 3.3 Sensing mechanism for the selective detection of the AMG class of antibiotics using AgNP–MZL

**Fig. 8:**
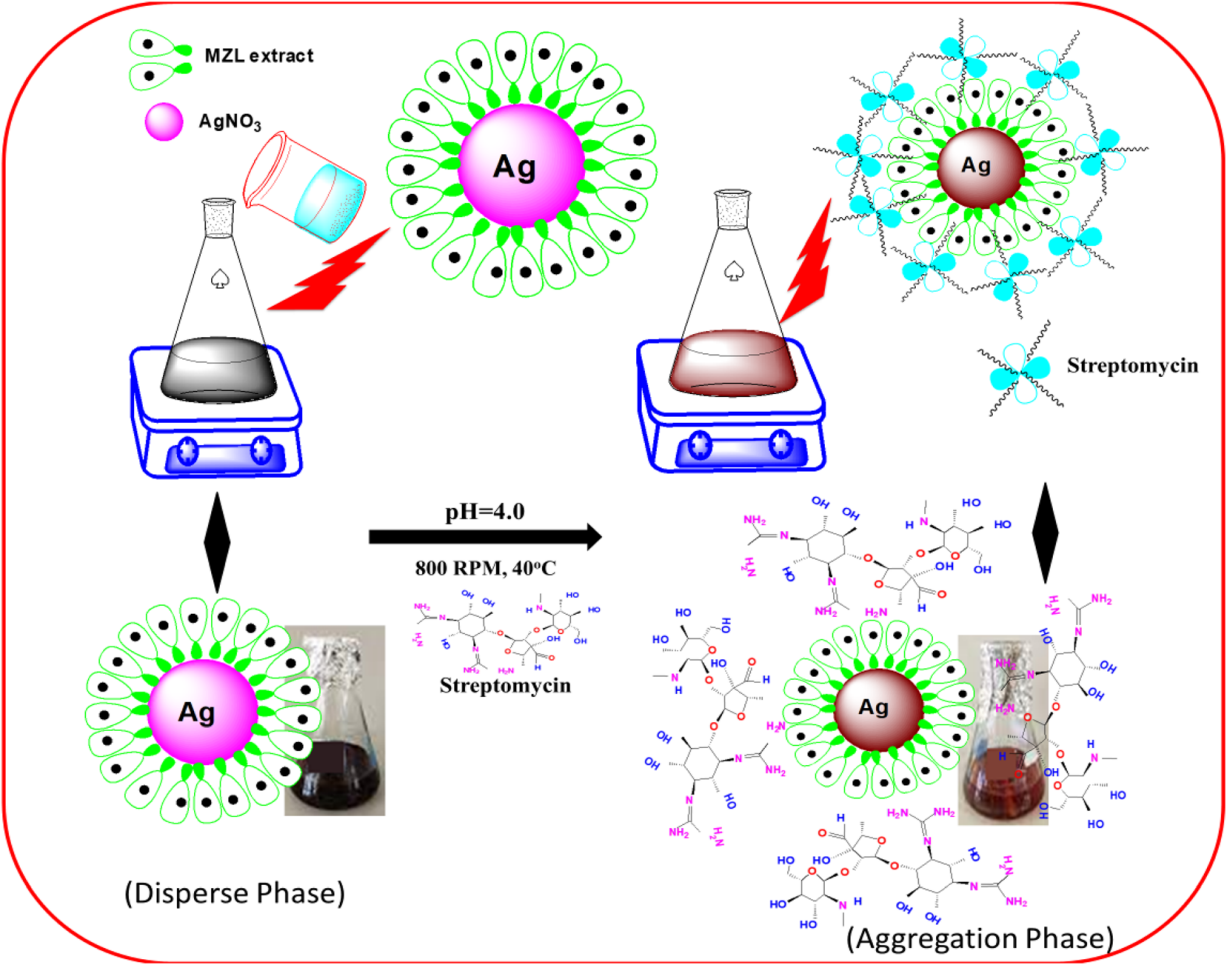
Sensing mechanism for the detection of AMG class of antibiotics using green synthesized AgNP–MZL.

To elucidate the mechanism of the activity between green synthesized AgNP–MZL and STR, several analytical techniques were used to monitor both physical and chemical changes in the AgNPs in the presence of antibiotics. In the present investigation, the colorimetric method is employed for the selective determination of the AMG class of antibiotics using green synthesized AgNP–MZL from different environmental, agricultural and soil samples. Different antibiotics, such as dicholorovos, thiochloprid, monocrotophos, cypermethrin, acetamethrin and bifenthrin, were chosen to demonstrate the selective determination of the AMG class of antibiotics with green synthesized AgNP–MZL. For this, all the antibiotics and AgNP–MZL were separately taken into a 10 mL glass vial in a volume ratio of 1 : 1 while maintaining the pH of the sample to 4.0 and kept at room temperature for 5 min of reaction time. The AgNP–MZL solution with antibiotics such as dicholorovos, thiochloprid, monocrotophos, cypermethrin, acetamethrin and bifenthrin showed an LSPR absorption peak at 390 nm, which was found similar to the UV-Vis spectrum of disperse AgNP–MZL (Fig. S1†). However, with the addition of STR into the solution of AgNP–MZL, the plasmon band at 390 nm shifted along with the appearance of a new peak at about 570 nm, Fig. 8 (S1). The color of the sample solution changed from dark blackish brown to dark brownish, and a shift in the LSPR absorption band was obtained due to the aggregation of the particles after the addition of STR only and not with other antibiotics. These results suggest that this biosensor, such as AgNP–MZL, displayed highly favorable and specific recognition of STR among common antibiotics. These results were confirmed by analyzing TEM, FTIR, XRD, DLS and zeta potential data and correlated with results obtained using reference methods.^30^ The results are shown in the above paragraph, *i.e.*, Results and discussion (Subsection 3.1).

The signal intensity of NPs was significantly reduced when STR was added onto the surface of AgNP–MZL, which could be attributed to the binding of STR onto the surface of the developed nanosensor. The phytochemicals in MZL extracts help in the reduction of Ag^+^ ions into Ag atoms, which subsequently combine to create AgNPs, as well as the stabilization of the NPs by preventing agglomeration. The negatively charged (−ve) phytochemical groups of the MZL extract might induce an electrostatic force of interaction with the positively charged (+ve) containing Ag^+^, significantly inhibiting redox probe electron transfer. The energy shift of these oxygen-containing functional groups to a higher level suggests electron donation. Consequently, complex formation between metal ions and AgNP–MZL occurred *via* the carbonyl and hydroxyl groups of the molecular moieties on their surfaces, resulting in AgNP agglomeration and, as a result, colour change. STR is challenging to detect because it lacks spectroscopic and electrochemical properties. This present method is based on the polycationic nature of the aminoglycoside (AMG), together with negatively charged (−ve) green synthesized AgNP–MZL. Most of the antibiotics that come under the class of the AMG carry a positive charge (+ve). The interaction of opposite charges between negatively charged AgNP–MZL and STR causes an aggregation-induced characteristic shift in the LSPR band of the AgNP–MZL probe in aqueous solutions. In the case of AgNP–MZL, the STR ligands bring the AgNP–MZL into close contact, causing the LSPR band to undergo a bathochromic shift in wavelength from 390 to 570 nm and a distinct visual colour change from dark blackish brown to dark brown. The amine functional groups of the STR act as a molecular linker, initiating the electrostatic coupling interactions among adjacent AgNP–MZL and, ultimately, driving the formation of well-defined AgNP–MZL aggregates.^37^ In addition, the lone pairs of electrons on the nitrogen atom of one primary amino group of the STR molecule attack the electron deficiency center. The sensing mechanism for the detection of STR using green synthesized AgNP–MZL is shown in Fig. 8. Based on this entire mechanism, STR was selected as a model compound for studying the broadening of the SPR band and its quantification in the ppb-level (ng mL^−1^).

### 3.4 Determining factors affecting the performance of the colorimetric method using AgNP–MZL

To obtain the ideal conditions for colorimetric detection of the AMG class of antibiotic (STR) using green synthesized AgNP–MZL, the combination concentration of nanoparticles, reaction time and effect of pH were determined. The effect of green synthesized AgNP–MZL concentrations at different stages in the range of 0.05–2.0 mM was optimized for the determination of STR in a colorimetric probe. The UV-Vis absorption band decreased gradually as the AgNP concentration increased from 0.05 to 2.0 mM, and when the concentration was 0.5 μM, the signal intensity almost reached the bottom. This indicates that the STR was binding on the surface of the AgNP–MZL. Therefore, 0.5 μM was the ideal concentration for the selective detection of STR in environmental, agricultural and soil samples. The results are shown in Fig. S2(a).† Images of glass vials containing AgNP–MZL after the addition of STR with different pH levels of the solution mixture and their UV-Vis absorbance bands are shown in Fig. S2(b).† The effect of pH on the interaction between AgNP–MZL and STR was studied over a pH ranging from 2.0 to 12, and the pH adjustment was performed by the addition of appropriate amounts of diluted HCl or NaOH solution. Thus, pH 4.0 was selected for further experiments. The effect of the reaction time was also investigated in the range of 1.0–10 min to determine the STR from the sample solution. The reaction time is also a key factor affecting the colorimetric results. It can be observed that the mean colour intensity gradually increased from 1.0 to 5.0 min and remained steady at 5.0 min, indicating that the etching of the green synthesis AgNP–MZL was completed within 5 min. Hence, 5 minutes was chosen as the reaction time for subsequent sensor detection. Therefore, the optimal condition was chosen for the following detection (Fig. S2(c)†). Ionic strength is another important parameter for improving the detection of the AMG class of antibiotics. The dependence of AgNP–MZL absorbance response in the presence of different concentrations of NaCl (5–50 mM) with a 5 min incubation was also investigated. This study is also vital to determine the effect of NaCl (50 mM) on the MZL extract-mediated AgNP probe against salt-induced aggregation. Aggregation of the AgNP–MZL occurred with the increasing addition of NaCl. The subsequent addition of streptomycin induced the MZL extract-mediated AgNP probe, followed by aggregation depending on the NaCl concentration. The results are shown in Fig. S2(d).†

### 3.5 Effects of potentially interfering compounds and cross-contaminants

To evaluate the selectivity of the sensing system for AMG antibiotics, *i.e.* STR detection, ionic compounds and other environmentally relevant antibiotics, including dicholorovos, thiochloprid, monocrotophos, cypermethrin, acetamethrin, bifenthrin and ionic compounds, were detected instead of the target under the same conditions. Selectivity describes whether an analytical method could discriminate the interference of similar groups of compounds in the presence of analytes, which is determined by the relative absorbance value in the colorimetric/AgNP method. For this, 1.0 mL standard antibiotics and 1.0 mL AgNP–MZL solution were added into known concentrations of organic and inorganic chemical species, and the matrix was analyzed by UV-Vis. The ratio of absorbance band intensities at 390–570 nm was used to assess the degree of AgNP–MZL aggregation in the colorimetric measurements. The results are shown in Fig. S3.† The absorption band of antibiotics remained unchanged even in the presence of the following tested organic and inorganic chemical species under the optimized condition of UV-Vis, while only STR displayed a decrease in color change from dark blackish brown to dark brownish as well as a red shift of LSPR absorption band. No influence was observed in the 1500-fold excess concentration of I^−^, Br^−^, Cl^−^, F^−^, CH_3_COO^−^, BrO_3_^−^, AsO_4_^3−^, MoO_4_^2−^, CrO_4_^2−^, and C_2_O_4_^2−^; 2000-fold excess concentration of ClO_2_^−^, BrO_3_^−^, IO_3_^−^, MnO_4_^−^, NO_2_^−^, NO_3_^−^, S_2_O_3_^2−^, WO_4_^2−^, SO_3_^2−^, CO_3_^2−^ and SO_4_^2−^; and 1450-fold excess concentration of Na^+^, K^+^, Mg^2+^, Ca^2+^, Ni^2+^, Zn^2+^, Fe^3+^, and Co^2+^. The other antibiotics, such as dicholorovos, thiochloprid, monocrotophos, cypermethrin, acetamethrin and bifenthrin, were also tested, and their presence in the samples did not interfere with the determination of antibiotics, such as STR, in agricultural and environmental samples. The tolerance limits of diverse ions and other antibiotics for the determination of STR using AgNP–MZL in environmental, agricultural and soil samples are shown in Table 2.

**Table 2:** Effect of diverse ions on the determination of AMG classes of antibiotics, such as STR, using green synthesis AgNP–MZL in environmental and agricultural samples

Diverse ions	Tolerance limit, mg L^−1^	
	Environmental sample	Agricultural samples
I^−^, Br^−^, Cl^−^, F^−^, CH_3_COO^−^, BrO_3_^−^	450	450
ClO_2_^−^, BrO_3_^−^, IO_3_^−^, MnO_4_^−^, NO_2_^−^	650	650
NO_3_^−^, S_2_O_3_^2−^, WO_4_^2−^, SO_3_^2−^, CO_3_^2−^, SO_4_^2-^	750	750
AsO_4_^3−^, MoO_4_^2−^, CrO_4_^2−^, C_2_O_4_^2-^	800	800
Na^+^, K^+^, Mg^2+^, Ca^2+^, Ni^2+^, Zn^2+^, Fe^3+^, Co^2+^	800	800
Dicholorovos	250	250
Thiochloprid	200	200
Monocrotophos	230	230
Acetamethrin	500	500
Bifenthrin	130	130
Cypermethrin	450	450

### 3.6 Analytical evaluation for the determination of AMG class of antibiotic using green synthesized AgNP–MZL

**Fig. 9:**
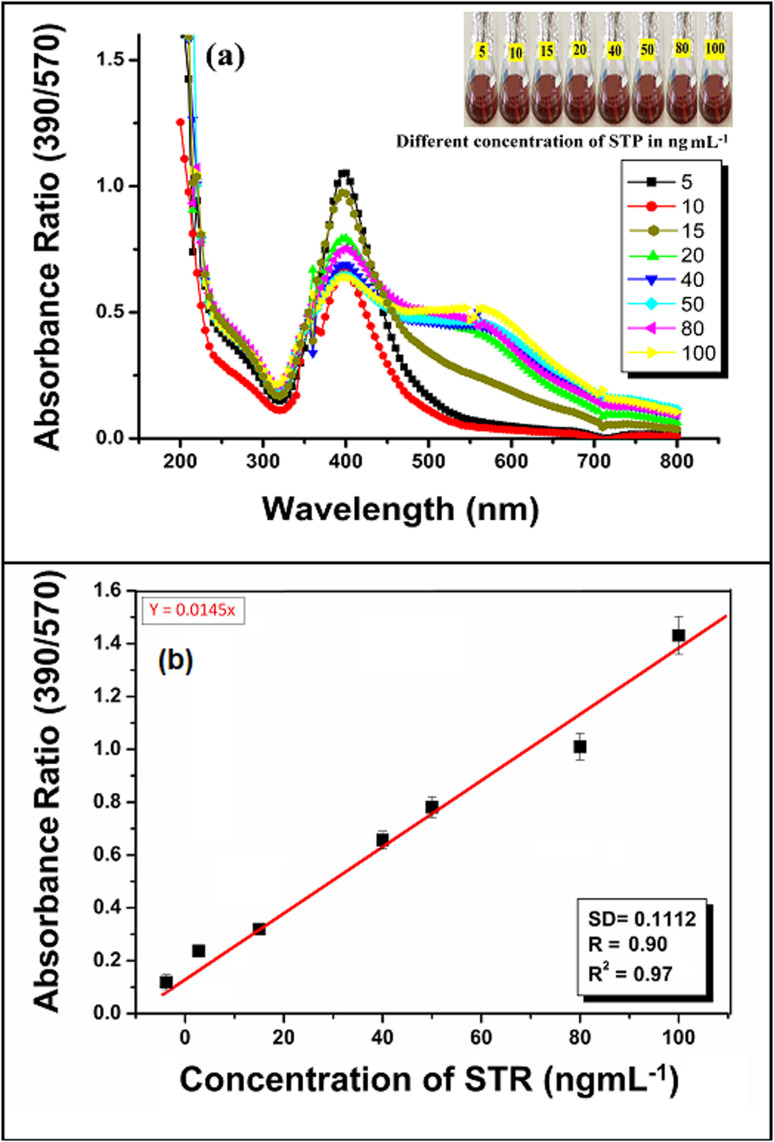
Photographic images of glass vials containing a solution mixture of AgNP–MZL with different concentrations of STR (5 ng mL^−1^, 10 ng mL^−1^, 15 ng mL^−1^, 20 ng mL^−1^, 40 ng mL^−1^, 50 ng mL^−1^, 80 ng mL^−1^ and 100 ng mL^−1^), with their UV-Vis spectra at pH 4.0 for 5 min reaction time at room temperature (a) and calibration curve for different concentrations of AMG class of antibiotics, such as STR (b).

Some important analytical parameters such as linearity range, limit of detection (LOD), limit of quantification (LOQ), correlation coefficient (*R*^2^), standard deviation (SD) and intraday–interday repeatability in terms of relative standard deviation (RSD), accuracy and precision for the determination of AMG antibiotic (STR) were evaluated to determine the plausibility of using green synthesized AgNP–MZL extract.^38^ According to the recognized STR detection scheme, the sensitivity of this established method was investigated. To test the sensitivity level of this method, the calibration curve was prepared for STR by adding different concentrations (5, 10, 15, 20, 40, 50, 80 and 100 ng mL^−1^) of antibiotics such as in different glass vials comprising 1.0 mL of AgNP–MZL at pH 4.0, and the total volume was maintained at 5.0 mL with ultrapure water. A colour change from dark blackish brown to dark brown was observed with a further increase in the intensity of the red-shifted band when the amount of STR was increased from 5 to 100 ng mL^−1^. The dependence of the ratiometric results (*A*_390_/*A*_570_) on the concentration of STR was expressed in a calibration plot in a range of 5–100 ng mL^−1^ of STR, which showed a coefficient of determination (*R*^2^) of 0.909. The results are shown in Fig. 9(a) and(b). The LOD was also evaluated by adding a minimum amount of STR into the AgNP–MZL solution using three times the SD with the slope of the curve (3SD/slope).^39,40^ LOQ is the lowest concentration of analyte at 10 × SD by acceptable precision at a similar concentration, *i.e.*, LOQ = 10SD/slope.^38^ The values of LOD and LOQ in this method were calculated to be 3.5 ng mL^−1^ and 26.8 ng mL^−1^, respectively. To the best of our knowledge, this is currently the most sensitive method for the colorimetric detection and quantification of STR in environmental and agricultural samples, with an LOD of about 3.5 ng mL^−1^. The precision of the method was obtained by calculating the intraday–interday repeatability in terms of relative standard deviation (RSD%) by three replicate analyses of the samples under the optimized conditions. The intraday-interday precision in terms of RSD was found to be 2.0% and 2.42% at 10 ng mL^−1^, showing a good precision of the method to determine STR in the sample solution (Fig. S4†), with relative standard deviations of 2.0% (intraday) and 2.42% (interday)^1^

Next, in the present work, the antioxidant activity of the aqueous extracts of MZL and AgNP–MZL was established through the entrapment of the DPPH free radical mechanism. This treatment procedure decreased the presence of DPPH in a concentration-dependent manner although they were less potent than bioactive compounds, such as quercetin, a reference antioxidant flavonoid.^41,42^ The DPPH scavenging activity of green synthesized AgNP–MZL was found to be 48.65% in this present research. Therefore, the AgNP–MZL showed a lower content of bioactive compounds (flavonoid) compared to the extract alone, which may correspond to the participation of these compounds in the reduction reaction involved in the biosynthesis of the AgNP–MZL from plant leaf samples. The results are shown in Fig. S5(a).† The antibacterial activity was assayed using the diameter of the inhibition zone formed around the AMG antibiotic and was used as a positive control with different bacterial strains, such as *E. coli*, *S. aureus*, and *E. faecalis*, using the disc diffusion method. The present study proves that AgNP–MZL shows relevant antibacterial activity against *E. coli* and *S. aureus*. The results of the antibacterial activity of MZL-mediated AgNPs are presented in Table 3 and Fig. S5(b) and (c).† The results showed that the zone of inhibition of bacterial growth on disc plates was a function of the different concentrations of AgNP–MZL. The growth of bacterial pathogens was inhibited gradually with an increase in the concentration of AgNP–MZL, and the results of this method were compared with the results obtained by the reference method using the standard antibiotic STR (Table 3). Parashar and Garg (2022) and Parashar and Garg (2023) reported that AgNPs synthesized by MZL possess significant antibacterial and antioxidant activity.^26,43^

**Table 3:** Antibacterial activity of green synthesized AgNPs from the leaf extract of *Manilkara zapota*^a^

Name of bacterial pathogens	Green synthesized AgNPs from leaf extract of *Manilkara zapota*			Standard antibiotic (STR)^a^
	Zone of inhibition (diameter, nm)			
	05 μg per disc	10 μg per disc	20 μg per disc	
*Escherichia coli*	10 ± 3.4	15 ± 1.2	19 ± 5.2	25 ± 4.6
*Enterococcus faecalis*	ND	ND	ND	ND
*S. aureus*	6. ± 1.9	10. ± 2.1	18. ± 1.2	20 ± 1.1

a ND = not detected; a = the disc with STR (20 μg per disc) was placed cultural plate for positive control.

### 3.7 Application of the determination of STR using AgNP–MZL in agricultural, environmental and soil samples

To investigate the practicality of the established STR detection method, the recovery study of this green synthesized AgNP–MZL-based biosensor was performed in four types of real-water samples, including surface water, river water, tap water, sewage water, and river water (SW, TW, SGW, and RW), four types of soil samples (SR-1, SD-2, SR-3, and SB-4) and three types of agricultural samples, including potato (Ag-P), tomato (AgAg–T) and green beans (AgAg–G). An aliquot of filtered water/soil/agricultural sample (1.0 mL) was added into a glass vial containing 1 mL of green synthesized AgNP–MZL at pH 4.0. The solution mixture was kept at a 5 min reaction time while maintaining the pH of the sample solution. AgNP–MZL and AgNP–MZL enriched with STR were directly used for colorimetric analyses. The analytical protocols for the analysis of real samples are described with a schematic diagram in the above-mentioned Experimental design section (Fig. 1). In the present work, the quality control experiment was carried out for each sample consisting of a linear calibration standard in a matrix, a blank and a spiked sample for the compounds. Two-spiked concentrations (10 ng mL^−1^, 50 ng mL^−1^) were chosen according to the sensitivity of our developed AgNP–MZL, and the selected concentrations were within the universal concentration range (ng mL^−1^) of antibiotics detected in Indian standard water/soil/agricultural samples (Jadhav *et al.*, 2013).^44^ The concentrations measured by this green synthesized AgNP–MZL biosensing platform were compared with spiked concentrations, and the results are presented in Table 4. When the non-spiked samples were poured into the green synthesized AgNP–MZL solution under optimized conditions, no significant change in the LSPR band was observed, indicating that STR was not detected in any samples. For spiked samples, both in the case of low and high STR concentrations, the calculated STR concentrations were close to the original spiked values. It was noted that the recovery rates in the water samples were lower than those in the other samples, such as soil and agricultural. In summary, the recovery rates of STR ranged from 67–107%, demonstrating the satisfactory accuracy of this green synthesized AgNP–MZL and indicating the application potential in real samples with a simple pre-treatment. The detection of the AMG class of antibiotic, *i.e.* STR, was possible visually at a concentration ranging from 15.6 ± 0.56 to 73.3 ± 0.38, which is far lower than the MRL set for AMG antibiotic, *i.e.*, STR by the European Union, China, and the World Health Organization.^45^

**Table 4:** Recovery percentage (%) for colorimetric detection of STR using green synthesized AgNP–MZL in agricultural and environmental samples

S. no.	Sample	Sample code	Standard addition (ng mL^−1^)	STR found (ng mL^−1^ ± SD)	RSD (*n* = 3), %	Error	Error, %	Recovery, (%)
1	Soil	SR-1	—	56.4 ± 0.47	0.4	—	—	—
			10	64.8	—	−8.40	−84.0	84.0
			50	109.1	—	−52.7	−105.4	105.2
		SD-2	—	53.2 ± 0.63	3.8	—	—	—
			10	63.6	—	−10.4	−104.0	104.0
			50	97.4	—	−44.2	−88.4	88.4
		SR-3	—	73.3 ± 0.38	2.0	—	—	—
			10	80.4	—	−7.10	−71.0	71.0
			50	115.2	—	−41.9	−83.8	83.8
		SB-4	—	62.0 ± 0.75	4.7	—	—	—
			10	71.2	—	−9.2	−92.0	92.0
			50	114.6	—	−52.6	−105.2	105.2
2	Water	SW	—	51.1 ± 0.40	3.3	—	—	—
			10	60.8	—	−9.70	−97.0	97.0
			50	103.6	—	−52.5	−105.0	105.0
		TW	—	51.9 ± 0.60	5.4	—	—	—
			10	58.6	—	−6.70	−67.0	67.0
			50	89.3	—	−37.4	−74.8	74.8
		SGW	—	55.2 ± 0.64	4.8	—	—	—
			10	62.7	—	−7.50	−75.0	75.0
			50	92.8	—	−37.6	−75.2	75.2
		RW	—	48.7 ± 0.40	3.6	—	—	—
			10	57.2	—	−8.50	−85.0	85.0
			50	99.8	—	−51.1	−85.2	85.2
3	Agricultural	AgAg–P	—	29.1 ± 0.31	4.7	—	—	—
			10	38.6	—	−9.50	−95.0	95.0
			50	76.1		−47.0	−94.0	94.0
		Ag-T	—	15.6 ± 0.56	3.6	—	—	—
			10	25.7	—	−10.1	−101.0	101.0
			50	69.1	—	−53.5	−107.0	107.0
		Ag-G	—	52.4 ± 0.21	3.6	—	—	—
			10	59.2	—	−6.80	−68.0	68.0
			50	92.1	—	−39.7	−79.4	79.4

Next, the linearity range and LOD values obtained by newly developed green synthesized AgNP–MZL were compared with other reported methods for the selective determination of STR, as shown in Table 5. The LOD value obtained by applying the present method was found to be comparable with colorimetry, electrochemistry, photoelectricity, fluorescence spectrometry, enzyme-linked immunosorbent assay, cyclic voltammetry and liquid chromatography-mass spectrometry.^45–52^ These earlier reported methods require time-consuming sample preparation procedures, trained personnel and high cost chemical reagents. The present method based on green synthesized AgNP–MZL is very simple, sensitive, selective, rapid, and cost effective, environmentally friendly and requires a minimum quantity of chemical reagents compared to column separation and chromatographic methods. In addition, compared with the reported achievements, the following advantages can be observed. In the present method, AgNP–MZL and colorimetry were combined to fully exploit the advantages of green nanotechnology and were applied to STR sensing detection (Table 5). It is applicable to various types of food and environmental field detection. Therefore, this sensor has a lower detection limit and a wider STR detection range, and the results can be trusted.

**Table 5:** Comparison of different analytical methods for STR detection

S. no.	Methods	Principle	Linearity range	LODs	Ref.
1	Colorimetric	Unmodified gold nanoparticles	0.2–1.2 μM	200 μM	45
2	Electrochemistry	Porous carbon nanorods graphene-based signal enhancement	0.05–300 ng mL^−1^	0.036 nM	46
4	Photoelectricity	CdTe quantum dot and single-wall carbon nanoglue	0.1–50 nM	0.033 nM	47
5	Fluorescence	Gold nanoparticles	1 × 10^−7^ to 0.0 M	47.6 M	48
6	Fluorescence	SPA-based evanescent wave	60–526 nM	33 nM	49
7	Enzyme-linked immunosorbent	Bead-based 96-well filtration plate competitive immunoassay	16–205 ng mL^−1^	Not given	50
8	Cyclic voltammetry	Electrochemical aptasensor	21.7–1087 ng mL^−1^	0.028 ng mL^−1^	51
9	Liquid chromatography-mass spectrometry	CdSe(*x*)Te(1 − *x*)/TiO_2_ nanotube structure-based label-free immunosensor	1.0–20 ng mL^−1^	2.0 ng mL^−1^	52
10	Colorimetric	Green synthesized AgNP–MZL	5–100 ng mL^−1^	3.5 ng mL^−1^	**Present work**

## 4 Conclusion

For the first time, a green synthesized AgNP–MZL sensing probe was reported for the rapid colorimetric detection of AMG antibiotics such as STR in agricultural and environmental samples. The polyphenols present in the MZL extract act as both reducing and capping agents for the synthesis of AgNPs. The sensitivity of the colorimetric method is based on the surface plasmon resonance (SPR) band changes and aggregation of green synthesized AgNP–MZL upon the successive addition of AMG classes of antibiotics, such as STR. The selectivity of the proposed sensing scheme towards STR in the presence of potentially interfering chemical substances and other classes of antibiotics was also demonstrated. The antibiotic serves not only as STR but also as a molecular linker for electrostatic coupling with the AgNP–MZL probe, yielding a noticeable colour change from dark blackish brown to dark brown, which can also be detected by UV-Vis spectroscopy. This AgNP–MZL also acts as an antioxidant and antibacterial activity compared to other green synthesized nanomaterials. Analysis of streptomycin in environmental, agricultural and soil samples was demonstrated, with excellent nanogram-level sensitivity. According to the feasible detection mechanism, the sensitivity detection of STR is achieved, including LOD and a wide detection range compared with other reported methods. The colorimetric method is simple, fast, cost-effective and selective; it does not require the use of huge amounts of organic solvents and does not require specific pre-treatment of samples.

## Conflicts of interest

The authors declare that they have no competing interests.

## Supplementary Material

RA-014-D4RA01906G-s001
