# Supplementary material for: Green synthesis of silver nanoparticles from Manilkara zapota leaf extract for the detection of aminoglycoside antibiotics and other applications
Source: RSC Adv. 2024 Jul 23;14(32):23240–56. doi: 10.1039/d4ra01906g (PMC11265568; doi:10.1039/d4ra01906g)
Supplement: RA-014-D4RA01906G-s001 [file RA-014-D4RA01906G-s001.pdf]

Electronic Supplementary Material (ESI) for RSC Advances.
This journal is © The Royal Society of Chemistry 2024

Supplementary Information

Green Synthesis of Silver Nanoparticles by *Manilkara Zapota* Leaf Extract for Detection of Aminoglycoside Antibiotic and its Applications

Khushboo Sahu[a], Ramsingh Kurrey[b*] and Ajai Kumar Pillai[a*]

[a]Govt. V.Y.T. Post Graduate Autonomous College Durg- 491001 Chhattisgarh, India

[b]National Center for Natural Resources, Pt. Ravishankar Shukla University Raipur-492010, Chhattisgarh, India

***Corresponding Author:**

Email: drajaipillai@gmail.com (Ajai Kumar Pillai),

Contact: +917882 393644

Figure caption

Fig. S1 Selectivity study: determination of AMG class of antibiotic like STR in the presence of other class of antibiotics with green synthesized AgNPs-MZL.

Fig. S2 Optimization parameters: effect of concentration of AgNPs-MZL (a), effect of pH (b), effect of reaction time (c) and effect of ionic salt (d).

Fig. S3 Effect of the diverse substances in the presence of the green synthesis AgNPs-MZL and AgNPs-MZL with AMG class of antibiotic for selective determination of STR.

Fig. S4 Reproducibility curve for determination of STR using green synthesized AgNPs-MZL at 10 $ngmL^{-1}$ under the optimized condition.

Fig. S5 The *in-vitro* antioxidant activity and antibacterial activity of the seed extract of *Manilkara Zapota,* green synthesized AgNPs-MZL and AgNPs-MZL with STR:

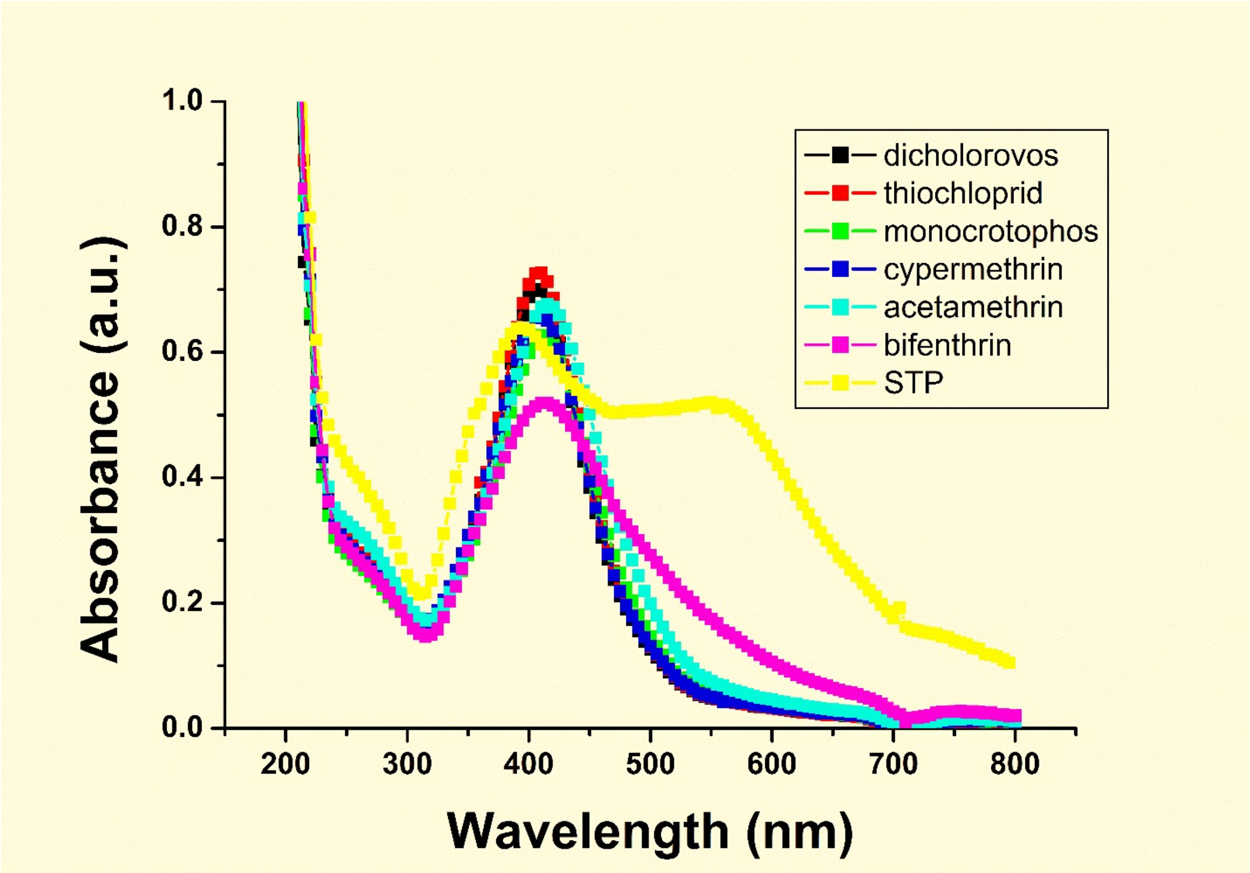
Absorbance (a.u.)
Wavelength (nm)
1.0
0.8
0.6
0.4
0.2
0.0
200
300
400
500
600
700
800
dicholorovos
thiochloprid
monocrotophos
cypermethrin
acetamethrin
bifenthrin
STP

Fig. S1

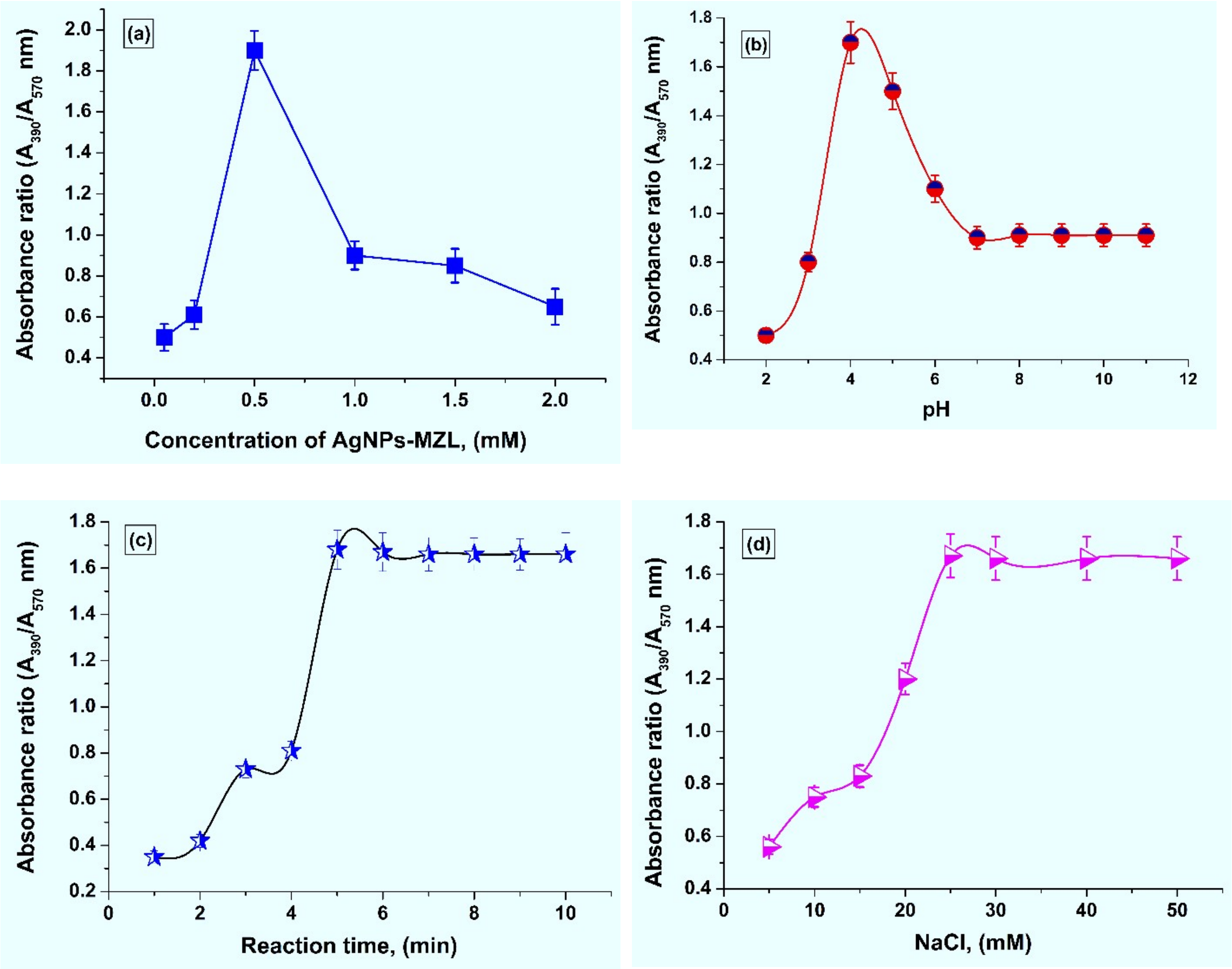
(a)
Absorbance ratio (A390/A570 nm)
Concentration of AgNPs-MZL, (mM)
(b)
Absorbance ratio (A390/A570 nm)
pH
(c)
Absorbance ratio (A390/A570 nm)
Reaction time, (min)
(d)
Absorbance ratio (A390/A570 nm)
NaCl, (mM)

Fig. S2

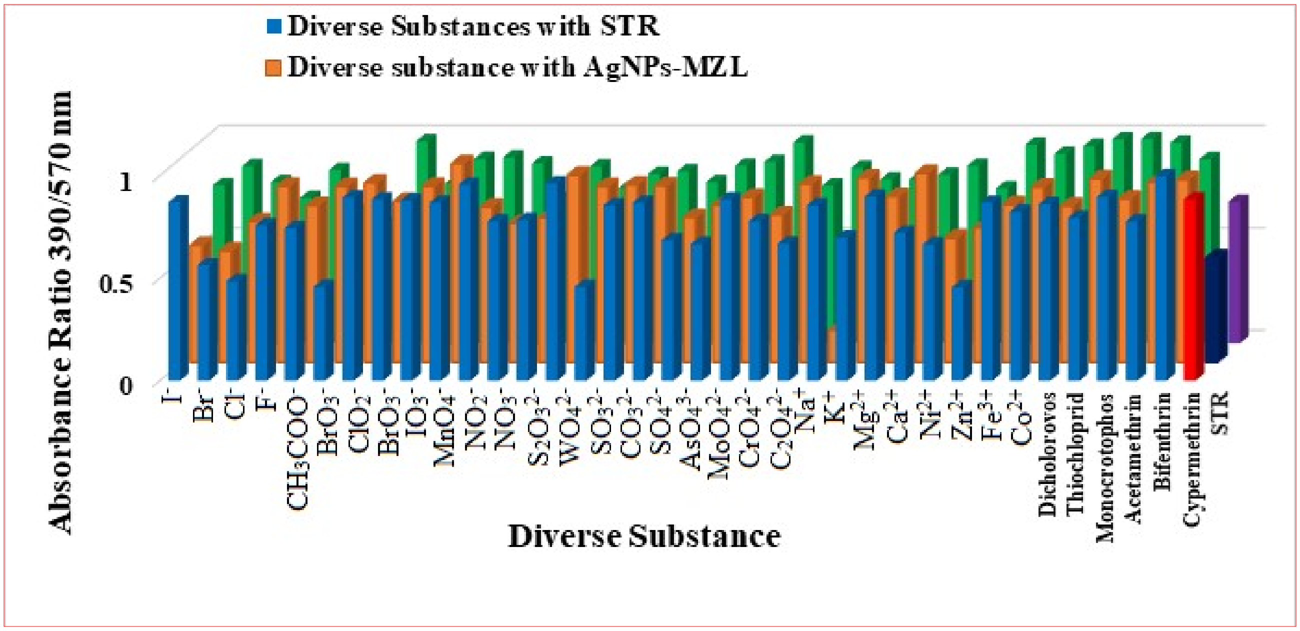

Diverse Substances with STR
Diverse substance with AgNPs-MZL
Absorbance Ratio 390/570 nm
1
0.5
0
Diverse Substance
Dicholorovos
Thiochloprid
Monocrotophos
Acetamethrin
Bifenthrin
Cypermethrin
STR

Fig. S3

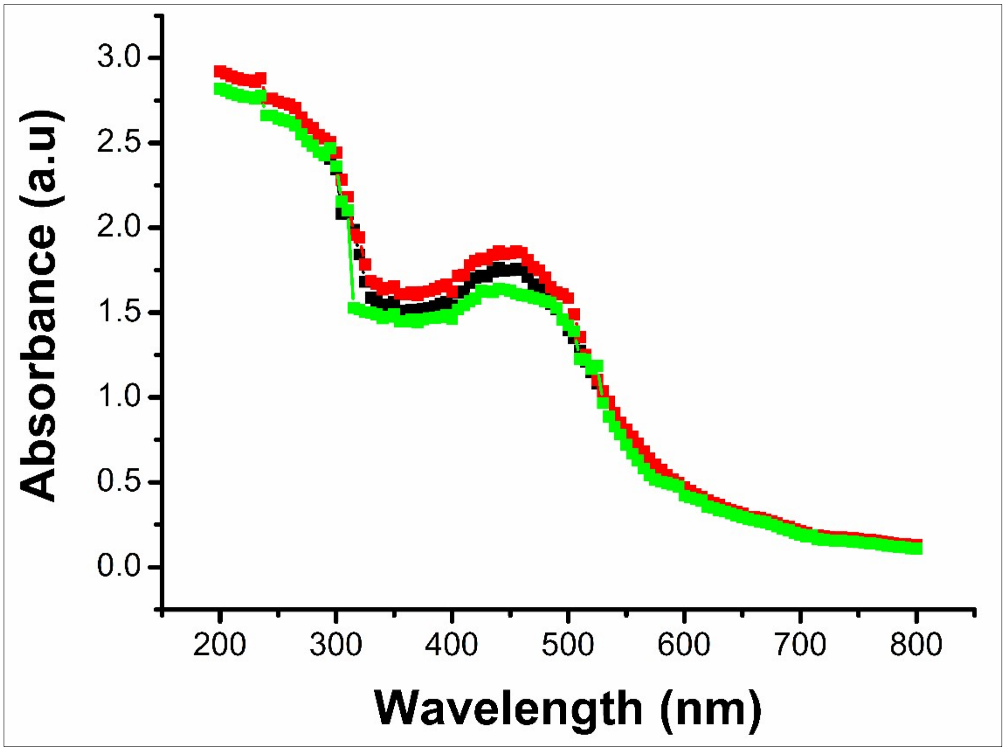

3.0
2.5
2.0
1.5
1.0
0.5
0.0
Absorbance (a.u)
200
300
400
500
600
700
800
Wavelength (nm)

Fig. S4

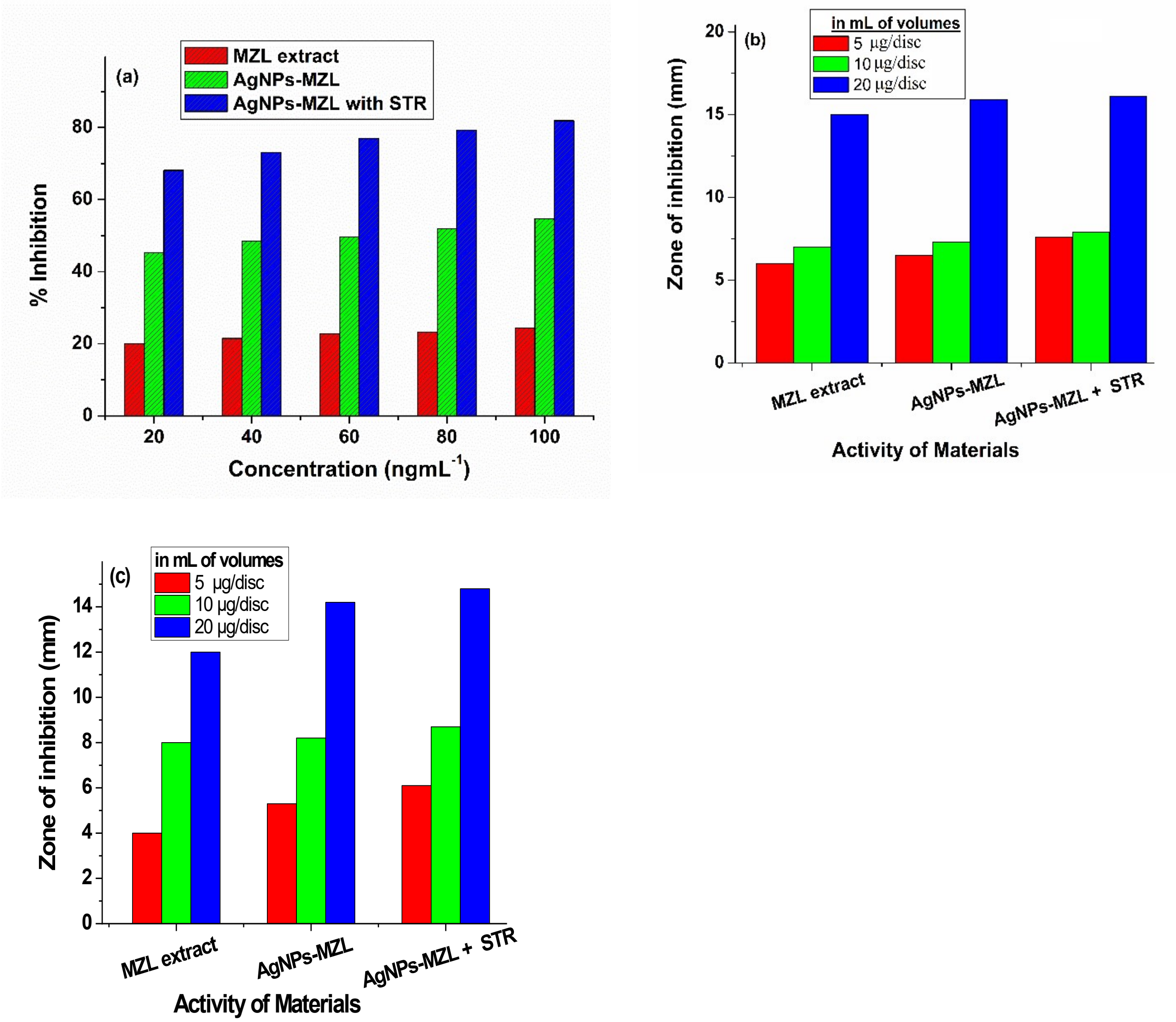
(a)
MZL extract
AgNPs-MZL
AgNPs-MZL with STR
% Inhibition
Concentration (ngmL-1)
0
20
40
60
80
100
(b)
in mL of volumes
5 µg/disc
10 µg/disc
20 µg/disc
Zone of inhibition (mm)
MZL extract
AgNPs-MZL
AgNPs-MZL + STR
Activity of Materials
(c)
in mL of volumes
5 µg/disc
10 µg/disc
20 µg/disc
Zone of inhibition (mm)
MZL extract
AgNPs-MZL
AgNPs-MZL + STR
Activity of Materials

Fig. S5
